# Missing and Possible Link between Neuroendocrine Factors, Neuropsychiatric Disorders, and Microglia

**DOI:** 10.3389/fnint.2013.00053

**Published:** 2013-07-15

**Authors:** Takahiro A. Kato, Kohei Hayakawa, Akira Monji, Shigenobu Kanba

**Affiliations:** ^1^Department of Neuropsychiatry, Graduate School of Medical Sciences, Kyushu University, Fukuoka, Japan; ^2^Innovation Center for Medical Redox Navigation, Kyushu University, Fukuoka, Japan; ^3^Department of Psychiatry, Faculty of Medicine, Saga University, Saga, Japan

**Keywords:** microglia, endocrinology, corticotropin-releasing hormone, glucocorticoids, sex hormones, estradiol, oxytocin

## Abstract

Endocrine systems have long been suggested to be one of the important factors in neuropsychiatric disorders, while the underlying mechanisms have not been well understood. Traditionally, neuropsychiatric disorders have been mainly considered the consequence of abnormal conditions in neural circuitry. Beyond the neuronal doctrine, microglia, one of the glial cells with inflammatory/immunological functions in the central nervous system (CNS), have recently been suggested to play important roles in neuropsychiatric disorders. However, the crosstalk between neuroendocrine factors, neuropsychiatric disorders, and microglia has been unsolved. Therefore, we herein introduce and discuss a missing and possible link between these three factors; especially highlighting the following hormones; (1) Hypothalamic-Pituitary-Adrenal (HPA) axis-related hormones such as corticotropin-releasing hormone (CRH) and glucocorticoids, (2) sex-related hormones such as estrogen and progesterone, and (3) oxytocin. A growing body of evidence has suggested that these hormones have a direct effect on microglia. We hypothesize that hormone-induced microglial activation and the following microglia-derived mediators may lead to maladaptive neuronal networks including synaptic dysfunctions, causing neuropsychiatric disorders. Future investigations to clarify the correlation between neuroendocrine factors and microglia may contribute to a novel understanding of the pathophysiology of neuropsychiatric disorders.

## Introduction

### Endocrine factors and neuropsychiatric disorders

Endocrine systems play an important role in bridging the body and the brain via various hormones, and maintaining homeostasis in physical conditions, while hormone imbalances are known to induce various physical disorders (Selye, 1950; Chrousos and Gold, 1992). Endocrine organs, such as the adrenal gland and the gonads, communicate with the central nervous system (CNS) via neuroendocrine factors and contribute to a series of mental functions. The network between the hypothalamus, pituitary, and adrenal gland, called the Hypothalamic-Pituitary-Adrenal (HPA) axis, is one of the crucial pathways in stress response (Selye, 1950; Chrousos and Gold, 1992).

The HPA axis is related to a variety of psychiatric conditions, such as the depressive symptoms associate with Cushing syndrome and steroid-induced depression (Lynn, 1995; Terao et al., 1997), suggesting that the dysregulation of the HPA axis is possibly involved with the pathophysiology of depression (Roy et al., 1988; Lesch et al., 1990). On the other hand, reproductive functions especially during pregnancy, birth, and the postnatal period are exerted exclusively by the gonads through sex-related hormones. Sex hormones establish the estrous cycle, and affect mental activities. The disturbance of estrous cycle functions is closely involved with various symptoms of mood and emotion, frequently developing into mood disorders such as premenstrual dysphoric disorder (PMDD) (Steiner et al., 1995; Epperson et al., 2002) and depressive disorders during perinatal (Bloch et al., 2000) and perimenopausal periods (Freeman et al., 2004a). Interestingly, oxytocin, the neuroendocrine hormone mainly associated with the postnatal period, has recently been highlighted for its stress-suppression effects and prosocial functions. Oxytocin has been shown to be involved with anxiety and depressive mood in patients with depression (Scantamburlo et al., 2007). Oxytocin exerts anxiolytic effects through the reduction of stress-induced corticosterone release on rodents (Windle et al., 1997) and also on healthy humans exposed to stress (Heinrichs et al., 2003). Additionally, oxytocin has been suggested to be an important factor in mental development and its abnormality may be related to autism (Tetreault et al., 2012). The above-mentioned reports all suggest that the HPA axis-related hormones, sex hormones, and oxytocin have a possible link to psychiatric disorders, while the underlying biological mechanisms have not been well understood.

### Novel understandings of neuropsychiatric disorders

Traditionally, abnormalities of neurons and neuronal networks including synaptic abnormalities and disturbance of neurotransmitters have dominantly been believed to be the main causes of psychiatric disorders. Beyond these classic understandings, novel theories have been presented that psychiatric disorders are systemic disorders widely driven by peripheral inflammatory and oxidative and nitrosative stress (O&NS) processes, and in addition that neuropsychiatric disorders are controlled by inflammatory, immune, O&NS, tryptophan catabolite, neuroinflammatory and neuroprogressive pathways in the CNS (Maes, 2011; Maes and Rief, 2012). Moreover, systemic inflammatory and O&NS processes heavily modulate hormonal levels, and reciprocal relationships between peripheral inflammation and hormonal levels are suggested to be involved in the pathophysiology of these disorders (Maes, 2011; Maes and Rief, 2012). In the brain, tryptophan catabolites such as kynurenine, kynurenic acid, 3-hydroxykynurenine, and quinolinic acid are synthesized in glia cells, and recent reports have shown that the blood kynurenine/tryptophan ratio is elevated in patients with depression and correlated with anxiety and cognitive disturbances (Schwarcz et al., 2012). Steiner et al. (2011) have shown pivotal findings that quinolinic acid positive microglia increase in the subgenual anterior cingulate cortex and anterior midcingulate cortex of patients with major depressive disorder (MDD) but not bipolar disorder. A relationship between the kynurenine pathway and depression is supposed to be induced by the activation of indoleamine 2,3-dioxygenase (IDO), an intracellular enzyme that catalyzes tryptophan degradation into kynurenine. However, how tryptophan catabolites correlate with depression pathology remains to be elucidated.

### Neuropsychiatric disorders and microglia

Macrophages in peripheral organs play important roles in the process of peripheral inflammation (Kiefer et al., 2001; Mosser and Edwards, 2008). Similarly, microglia, one of the immune cells in the brain, play crucial roles in neuroimmunology including neuroinflammatory and neuroprogressive pathways. Microglia were initially discovered by Del Rio-Hortega (1919). Traditionally, neuroscientists regarded microglial function simply to provide physical support and maintenance for neurons. Thus, microglia had been long ignored (Miller, 2005). However studies during the last 20 years have elucidated various biological functions of microglia that act as “brain macrophage”; crucial immunological/inflammatory players moving around, monitoring micro-environmental changes, and once activated, releasing pro-inflammatory cytokines such as tumor necrosis factor (TNF)-α and free radicals including nitric oxide (NO), and finally inducing neuropathologies such as phagocytosis, apoptosis of neuronal cells, suppressing neurogenesis, and oligodendrocyte dysfunction (Block et al., 2007; Hanisch and Kettenmann, 2007). Thus, microglia have proved to play important roles in neurodegenerative diseases and neuropathic pain (Inoue and Tsuda, 2009; Graeber, 2010; Graeber and Streit, 2010; Kettenmann et al., 2011; Ransohoff and Stevens, 2011).

Recent positron emission tomography (PET) imaging of peripheral benzodiazepine receptors and postmortem studies have suggested microglial activation in the brain of patients with neuropsychiatric disorders such as schizophrenia, depression, and autism (Steiner et al., 2006, 2008; van Berckel et al., 2008; Doorduin et al., 2009; Morgan et al., 2010; Takano et al., 2010; Tetreault et al., 2012; Suzuki et al., 2013). Interaction between microglial activation and psychopathology has not been well clarified, while a PET study has resulted in an interesting outcome; Takano et al. (2010) reported that chronic schizophrenia patients showed a positive correlation between cortical [11C]DAA1106 binding (one of the peripheral benzodiazepine receptors ligand) and positive symptom scores which were assessed using the Positive and Negative Syndrome Scale. Although the correlations need to be interpreted very cautiously, involvement of microglial activation in the pathophysiology of positive symptoms of schizophrenia might be suggested. Animal models of neuropsychiatric disorders such as schizophrenia and autism have proposed the underlying microglial pathologies (Juckel et al., 2011; Derecki et al., 2012; Liaury et al., 2012). In addition, various psychotropic drugs, which had classically been regarded to modulate solely neurons and synaptic networks, have recently been revealed to have direct anti-inflammatory properties on activated microglia in a series of *in vitro* studies (Hashioka et al., 2007; Kato et al., 2007, 2008, 2011b; Horikawa et al., 2010). Therefore, microglia may play crucial roles in the pathophysiology and treatment of neuropsychiatric disorders (Monji et al., 2009, 2013; Kato et al., 2011a, 2013).

In addition, minocycline, a tetracycline antibiotic, has recently been known to improve symptoms of psychiatric disorders such as schizophrenia (Miyaoka et al., 2007; Miyaoka, 2008; Levkovitz et al., 2010). Minocycline has a variety of functions in the CNS such as interacting with brain glutamate and dopamine neurotransmission (Kim and Suh, 2009) and having direct effects on neuronal cells (Hashimoto and Ishima, 2010). Rodent studies have revealed that minocycline inhibits microglial activation (Yrjanheikki et al., 1998), and in actuality it is one of the most frequently used drugs for inhibiting microglial activation (Yrjanheikki et al., 1999; Du et al., 2001; Kim and Suh, 2009). Several rodent studies have shown that stress increases microglial activation (Frank et al., 2007; Sugama et al., 2009; Tynan et al., 2010), and causes anxiety-like behaviors, which in turn can be decreased by minocycline treatment (Neigh et al., 2009). These *in vivo* studies have suggested that minocycline may be effective for the treatment of psychiatric disorders. An open-label study has shown that selective serotonin reuptake inhibitor (SSRI) and minocycline attenuate depressive and psychotic symptoms in patients with psychotic depression (Miyaoka et al., 2012). In addition, minocycline has been reported to be effective for the treatment of various symptoms in patients with Fragile X syndrome (FXS) such as social communication, anxiety, irritability, stereotypy, hyperactivity, and inappropriate speech (Paribello et al., 2010; Utari et al., 2010).

Previous microglia research has mainly highlighted the neuropathological aspects of microglia, while recent animal studies have shown the normal functions of microglia (Graeber, 2010; Ransohoff and Stevens, 2011; Tremblay et al., 2011; Graeber and Christie, 2012; Schafer et al., 2012). Rodent microglia have been revealed to monitor synaptic reactions by having continuous direct contact with synapses not only in pathological brain but also in normal brain (Wake et al., 2009), and have proved to play essential roles in brain development such as in synaptic pruning (Paolicelli et al., 2011; Schafer et al., 2012). Therefore, even in normal conditions, microglia have been revealed to have some crucial roles in the homeostasis of synaptic conditions and in brain development. Moreover, we have recently reported that human social activities such as decision-making are modulated by minocycline not only in psychiatric patients but also in healthy persons (Kato et al., 2012; Watabe et al., 2012). Our human neuroeconomics studies have implied the possibility that brain development including neuron-microglia network establishment may formulate personality, and personality-oriented behaviors may be modulated by microglia (Kato et al., 2012). Thus, we suppose that microglia could be one of the crucial players in human mental development during early stages, and also in various social/mental activities after developmental stages including under healthy and pathological conditions beyond the neuron-synapse doctrine. A recent PET study has shown that minocycline inhibits microglial activation in humans (Dodel et al., 2010), thus we can prospect that minocycline may actively modulate human microglial activity.

Based on the above-mentioned reports, we hypothesize that microglia may be one of the bridging players between these highlighted neuroendocrine factors, normal/pathological mental conditions, and psychiatric disorders, while the underlying biological mechanisms have yet to be well considered. In addition, it is unknown whether microglia release neuroendocrine factors, while recent studies have suggested that these hormones affect microglia. Therefore, in this article, we would like to introduce and discuss the missing and possible link between the above-mentioned neuroendocrine factors, neuropsychiatric disorders, and microglia.

## Hypothalamic-Pituitary-Adrenal Axis-Related Hormones

The HPA axis is the major stress response system which connects peripheral organs and the brain (Selye, 1950; Chrousos and Gold, 1992). In response to stress, corticotropin-releasing hormone (CRH) is secreted by the paraventricular nucleus (PVN) of the hypothalamus, and released into the pituitary gland, where CRH induces the release of adrenocorticotropic hormone (ACTH). Thereafter, ACTH activates the adrenocortical secretion of glucocorticoids. Finally, glucocorticoids suppress the release of CRH and ACTH as a negative feedback.

### HPA axis and psychiatric disorders

A variety of psychiatric symptoms due to primary physical illness are related to the HPA axis, such as the depressive symptoms associate with Cussing syndrome and steroid-induced depression (Lynn, 1995; Terao et al., 1997). The significant higher concentration of CRH and ACTH, induced by the initial injection of interferon (IFN)-α, has been found in patients with the subsequent development of depression during IFN-α treatment than in those without depression (Capuron et al., 2003).

The HPA axis has also been suggested to have a strong linkage to psychiatric disorders such as mood disorders and post-traumatic stress disorder (PTSD) (Baker et al., 1999; Holsboer, 2000; Kunugi et al., 2006). Baker et al. (1999) reported that CRH levels in cerebrospinal fluid were significantly greater in PTSD patients than in normal subjects. The dysregulation of the HPA axis is suggested to be involved with the pathophysiology of depression (Roy et al., 1988; Lesch et al., 1990). Previous studies have revealed the blunted responses to stressful events in patients with MDD, especially patients with a familial history of mood disorders and with men compared to women (Peeters et al., 2003). Investigations with depressed patients have shown that the number of CRH neurons increases in the PVN of the hypothalamus of patients with depression (Raadsheer et al., 1994), and pituitary volume decreases in patients with bipolar disorder (Sassi et al., 2001). ACTH has been reported to induce depressive-like behaviors through NMDA receptors in rats (Tokita et al., 2012). Glucocorticoids are also thought to be involved in various psychiatric disorders and related emotional disturbances (Brown, 2009; Laan et al., 2009; Ros-Bernal et al., 2011). Recently, Niwa et al. have reported the essential role of glucocorticoids in the association between adolescent stress and gene-environmental interactions using a mouse model with dominant-negative DISC1 (Disrupted Schizophrenia 1) under isolation during adolescence, and suggested this mouse as a candidate model for psychotic depression. They have revealed that via the epigenetic functions of glucocorticoids, adolescent stress induces projection, originating from the ventral tegmental area, specific methylation of tyrosine hydroxylase, and subsequently causes some neurochemical and behavioral deficits in this model mouse (Niwa et al., 2013). These reports have suggested that the HPA axis is one of the key components in understanding the deeper mechanisms of mood disorders and other psychiatric disorders.

### Corticotropin-releasing hormone and microglia

Corticotropin-releasing hormone, otherwise known as corticotropin-releasing factor (CRF), is a 41-amino acid peptide hormone, originally derived from a 191-amino acid preprohormone (Vale et al., 1981). The main site of CRH production is neurons in the parvocellular division of the hypothalamic PVN. CRH is also distributed in the limbic system such as other areas of the hypothalamus and amygdala, the locus ceruleus of the brain stem, A1, A5 catecholaminergic cell groups, and cerebral cortices (Chappell et al., 1986; Dunn et al., 2004; Chandrasekar et al., 2007). CRH mainly binds to CRH receptor I (CRH-R1). Urocortin, which also binds to CRH-R1, was discovered in rat midbrain (Vaughan et al., 1995), and is now characterized in three subtypes as urocortin-I/II/III (Lewis et al., 2001; Pelleymounter et al., 2004). Urocortins have high affinity to another CRH receptor; CRH receptor II (CRH-R2). Urocortin-II and urocortin-III specifically bind to CRH-R2. Urocortins are known to modulate various aspects; for example, appetite and anxiety in the brain, and the cardiovascular system in peripheral organs (Oki and Sasano, 2004).

Rat microglia have functional CRH-R1, and CRH has shown to bind to CRH-R1 (Wang et al., 2002), which results in microglial proliferation and TNF-α release via mitogen-activated protein kinase (MAPK) signaling pathways (Wang et al., 2003). On the other hand, Ock et al. reported that CRH induced an apoptosis of mice microglia, and did not influence NO production or expression of pro-inflammatory genes, indicating that CRH did not affect the inflammatory activation of microglia. The CRH-induced microglial apoptosis has shown to involve a mitochondrial pathway and reactive oxygen species (ROS), based on the mitochondrial membrane potential change, caspase 9 activation, and sensitivity to antioxidants (Ock et al., 2006). These reports have suggested that CRH induces a part of pro-inflammatory reactions and/or oxidative stress, while further investigations are needed for a more detailed understanding.

Interleukin (IL)-18 has been suggested to be one of the crucial cytokines modulating stress responses (Tringali et al., 2005). Microglia is the major source of IL-18 in the brain (Prinz and Hanisch, 1999). Recent reports have suggested that IL-18 may play a significant role in psychiatric disorders such as depression and PTSD (Shirts et al., 2008; Mehta et al., 2011; Prossin et al., 2011; Xiu et al., 2012). Yang et al. (2005) reported that CRH enhanced IL-18 mRNA expression and significantly induced the secretion of functional IL-18 protein in mouse BV2 microglial cells. IL-18 knockout mice showed less microglial activation after acute stress, which resulted in less damage of dopamine neurons (Sugama et al., 2004, 2007). On the other hand, CRH increased the generation of intracellular reactive oxygen intermediates (ROI), and CRH-induced IL-18 production was blocked by an antioxidant, *N*-acetyl-l-cysteine (NAC) in mouse microglia (Yang et al., 2005). This report suggests that stress response is involved in regulating CRH-induced IL-18 production via ROI modification in microglia.

Meanwhile, microglia in rat/mice also have functional CRH-R2 receptors (Wang et al., 2007). Urocortin suppressed the release of TNF-α from lipopolysaccharide (LPS)-activated microglia via CRH-R2, and attenuated the LPS-induced neuronal damage on neuron-microglia mix culture (Wang et al., 2007). This report indicates the possibility that urocortin has neuroprotective properties via anti-inflammatory effects on microglia, similar to other immunological cells (Gonzalez-Rey et al., 2010). A recent study using a triple urocortin knockout mouse model has suggested that urocortins play an essential role in stress recovery (Neufeld-Cohen et al., 2010). The latest study has revealed that CRH and urocortin modulate spinal outgrowth and synaptic formation via CRH receptors (Gounko et al., 2013). Direct interactions between microglia and these functions of CRH and urocortins have not been investigated (Neufeld-Cohen et al., 2010; Gounko et al., 2013), while we suppose that microglia may play an important role.

### Glucocorticoids and microglia

To our knowledge, a direct relationship between ACTH and microglia has not been reported. On the other hand, glucocorticoids, another key modulator of the HPA axis, are suggested to induce microglial modulation in the CNS (Sorrells et al., 2009). Previous studies have discussed the role of glucocorticoids as moderators of stress-related neuroinflammation (Dinkel et al., 2003; Munhoz et al., 2010; Loram et al., 2011). Smith et al. (1991) reported that adrenalectomy powerfully potentiates CNS inflammatory responses. Regarding microglia, Tanaka et al. (1997) have reported that cultured microglia isolated from the forebrain of newborn rats express glucocorticoid receptor in the plasma membrane. Sierra et al. (2008) have shown that corticosterone attenuated the production of TNF-α, IL-6, and NO from LPS + IFN-γ-activated murine microglia, which suggests that the anti-inflammatory effect of glucocorticoids on microglia is inverted to that of CRH. The stress-induced elevation of glucocorticoids has been well known (Munck et al., 1984), and it has proven to activate microglia in rats (Sugama et al., 2007; Tynan et al., 2010) and promote the proliferation of microglia via NMDA receptor activation in mice (Nair and Bonneau, 2006). From the temporal point of view it is important to categorize stress into two types; acute and chronic stress (Frank et al., 2010). Stress and administration of glucocorticoids prior to the injection of LPS, a peripheral immune stimuli, exerts pro-inflammatory effects on microglia in rats (de Pablos et al., 2006; Frank et al., 2010, 2012). On the contrary, exposure to glucocorticoids after LPS stimulation has anti-inflammatory properties in rats (Frank et al., 2010). In macrophage, glucocorticoids have been reported to exert pro-inflammatory effects through the increased NOD-like receptor (NLR) P3 mRNA and protein, which is a critical component of the inflammasome (Busillo et al., 2011). Deeper mechanisms of pro- and/or anti-inflammatory effects of glucocorticoids on microglia have not been well clarified, while a recent study has suggested that corticosteroids limit microglial activation occurring during acute stress when using adrenalectomy plus corticosterone administered rats (Sugama et al., 2013). Further investigations should be conducted to dig up the deeper mechanism.

### Clinical implications

**Figure 1:**
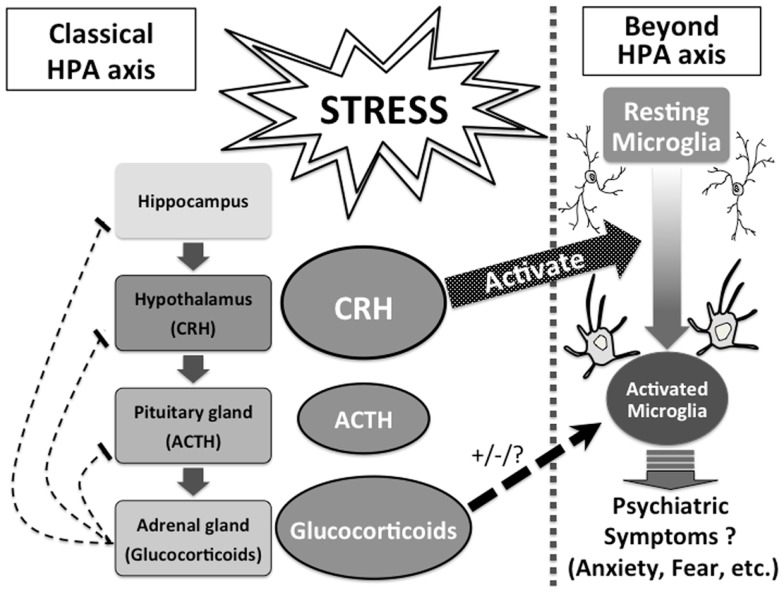
**CRH and glucocorticoids affect microglia beyond the HPA axis**.

The HPA axis has long been suggested to have strong linkage to psychiatric disorders, and the role of hormones such as CRH and glucocorticoids have been considered within the understanding of the HPA axis. Our highlighted evidence that CRH and glucocorticoids directly affect microglia sheds new light on understanding the unsolved roles of CRH and glucocorticoids in psychiatric disorders and psychopathologies. CRH and glucocorticoids directly and/or mutually modulate activities of microglia, and activated microglia release pro-inflammatory mediators, which may result in various psychiatric symptoms such as anxiety, fear, and depression beyond the classical understanding of the HPA axis (Figure 1). So far, the underlying mechanism of the mutual effects of CRH and glucocorticoids on microglia has not been well understood, while such novel knowledge will provide a systematic understanding of psychiatric disorders. Further basic, clinical and translational studies of CRH and glucocorticoids focusing on microglia should be investigated.

## Sex Hormones

Sex hormone system, frequently conceptualized as the hypothalamic-pituitary-gonadal (HPG) axis, is one major category of hormones correlated with mental conditions. Sex hormones such as estrogen, progesterone, and testosterone have crucial roles in health. Estrogen is one of the important steroid hormones and mainly classified into estrone, estradiol, and estriol. Androstenedione and testosterone metabolized from cholesterols in theca cells can be aromatized to estrone and estradiol in granulosa cells, respectively. Estradiol is also produced in various regions such as the adrenal gland, the mammary gland, adipose tissues, and the brain. From puberty to menopause, estrogens establish and maintain the reproductive function during the estrous cycle. In addition to these functions, estrogens have systemic functions that include for example, developing the mammary gland, regulating the immune system, protecting the cardiovascular system, and maintaining bones. At the same time, the remarkable involvement of estrogens in the brain have recently been highlighted, and the mechanism underpinning these functions such as enhancing cognitive function has gradually been clarified (Walf et al., 2011). Progesterone, one of the steroid hormones from the corpus luteum, is involved in the female reproductive system, especially developing the mammary gland and the initiation and maintenance of pregnancy (Mani and Portillo, 2010). Androgens are steroid hormones that are essential not only to the establishment and maintenance of male reproductive functions but also to muscular and bone anabolic functions (Yin et al., 2003). The most primary androgen is testosterone secreted by Leydig cells in the presence of luteinizing hormone (Mooradian et al., 1987). During reproductive age, sex hormones levels alter along with life stages of women. Estrogen and progesterone progressively increase during pregnancy, and after the delivery, both hormones abruptly decrease and a normal estrous cycle is restored. Also, both hormones gradually begin to decrease from the climacteric period toward menopause, but LH and FSH simultaneously increase during this period. In this way, sex hormones in women demonstrate cyclic alteration along with ovulation, and also the balance of sex hormones changes at the time of pregnancy or menopause, causing some women to show various psychiatric symptoms which occasionally develop into psychiatric disorders (Steiner et al., 2003).

### Sex hormones, mental conditions, and psychiatric disorders

Women are more likely to show unipolar depression than men (Nolen-Hoeksema, 1987), and severe stresses are more likely to induce depression in women (Kendler et al., 1995). Sex hormones alter with time and subsequently establish the estrous cycle, which plays an important role in depression.

Neurosteroids such as pregnenolone, progesterone, and dehydroepiandrosterone (DHEA) have been suggested to have positive links to neuropsychiatric disorders. For example, the CSF level of pregnenolone is lower in patients with MDD than healthy controls (George et al., 1994). The saliva and serum level alterations of DHEA during depressive episodes have revealed divergent results (Eser et al., 2006), but recently Kurita et al. (2013) have reported elevated levels of serum DHEA in male and female patients with MDD. Both pregnenolone and DHEA probably have therapeutic effects for patients with MDD (Wolkowitz et al., 1999; Schmidt et al., 2005; Osuji et al., 2010). Considering the binding abilities of pregnenolone, DHEA/DHEA-sulfate (DHEA-S), progesterone, and testosterone to sigma-1 receptors, and of DHEA-S and progesterone to GABAA receptors, these receptors may be a possible link between neurosteroids and neuropsychiatric disorders (Hashimoto, 2013).

#### Altered mental conditions during premenstrual period

Sex hormones are widely known to influence mental conditions. Many women with existing psychiatric disorders complain of worse mental symptoms before menstruation and experience various physical and mental symptoms of premenstrual syndrome. Some women with PMS show severe symptoms and are psychiatrically diagnosed with PMDD under the Diagnostic and Statistical Manual of Mental Disorders, Fourth Edition (DSM-IV) (Steiner, 2000). From the latter luteal phase to the just beginning of menstruation patients with PMDD show various mental symptoms such as depressive mood, anxiety, irritability, and insomnia. The levels of estrogen and progesterone drastically alter during the same period, suggesting that the alteration of sex hormones levels may be associated with the pathology of PMDD. Administering progesterone increases the reactivity of the amygdala in women with no symptoms during the follicular phase (van Wingen et al., 2008) and causes various physical and mental symptoms in postmenopausal women (Hammarback et al., 1985; Magos et al., 1986), suggesting that progesterone induces various psychiatric symptoms in women. However, the direct association between the level of progesterone and PMS/PMDD has not been confirmed (Rubinow et al., 1988; Schmidt et al., 1991). Patients with PMS/PMDD may be vulnerable to the fluctuation of the levels of estrogen and/or progesterone. Some reports using acoustic startle response or prepulse inhibition have shown that the response to the fluctuation of sex hormones differs between healthy women and patients with PMDD (Kask et al., 2008). Animal studies have suggested that the alterations of the level of estrogen/progesterone are positively associated with aggression (Ho et al., 2001) and the depressive trend (Schneider and Popik, 2007).

#### Altered mental conditions during postpartum period

Both estrogen and progesterone levels increase during pregnancy, and abruptly decrease after delivery. A few days after childbirth, many women show mild depressive mood, tearfulness, anxiety, irritability, dysphoria, and insomnia; generally referred to as “maternity blues” and considered to be a transient psychological response (Newport et al., 2002). Maternity blues is also a risk factor for depression and anxiety disorder within 3 months after delivery (Reck et al., 2009). Psychiatric disorders including depression are well known to appear frequently during the perinatal period, and estrogen and progesterone are suggested to be involved with the development of postnatal depression (Bloch et al., 2000). Freeman et al. (2002) have reported that 67% of women with bipolar disorder experience a postpartum mood episode, emphasizing the problem of the relapse and recurrence of a mood episode. Postpartum psychosis is a rare psychiatric disease characterized by hallucinations, delusions, mood swing, etc (Brockington et al., 1981). A lower blood concentration of estradiol is observed in patients of postnatal psychosis compared to normal controls, therefore the administration of estradiol could be effective to patients who are resistant to neuroleptic medications (Ahokas et al., 2000). Moreover transdermal estrogen replacement therapy has been reported to be an effective treatment of severe postnatal depression (Gregoire et al., 1996).

#### Depression during the menopausal transition

Sex hormonal changes and the estrous cycle become gradually irregular during menopausal transition, consequently causing climacteric syndromes including such physical symptoms as vasomotor symptoms (hot flashes and night sweat), and vaginal dryness, and mental symptoms such as depression and irritability (Anon, 2005). A controversial issue is that the risk for depression may be high during the periods of premenopausal, perimenopausal, and/or postmenopausal. Women with a history of PMS have been reported to be susceptible to depression during the perimenopausal period (Freeman et al., 2004b). Woods and Mitchell (1996) have suggested the association between consistent depressive symptoms during the perimenopausal period and a history of postpartum depressive symptoms. Morrison et al. (2004) have reported that hormonal replacement therapy is effective for women with postpartum and premenopausal depression, but not for women with postmenopausal depression. The mechanism of the pathogenesis of psychiatric disorders during menopausal transition remains controversial possibly because of the few studies utilizing rigorous measurements such as The Structured Clinical Interview for DSM-IV (SCID). By utilizing the SCID for women without a current or past history of depression, Schmidt et al. (2004) have revealed that the risk for onset of depression is 14 times as high as for a 31-year premenopausal period of time. In the Study of Women’s Health Across the Nation (SWAN), Bromberger et al. (2009) initially reported no association between the perimenopausal period and the risk for onset of depression diagnosed by SCID, but afterward, they have reported that the risk of major depression is greater for women during and immediately after the menopausal transition than for women during the premenopausal period (Bromberger et al., 2011). A higher blood concentration of testosterone is found in untreated premenopausal women with depression compared to normal controls (Baischer et al., 1995). A lower blood concentration of estradiol during the follicular phase and a shorter half-life of luteinizing hormone during both the follicular and luteal phase are found in premenopausal women with depression (Young et al., 2000). There is a significant negative correlation between the blood concentration of estradiol and Hamilton depression scores in depressed premenopausal women (Baischer et al., 1995). During transition to the menopausal period, women suffering from depressive symptoms increase, and contrastively, these symptoms decrease after menopause (Freeman et al., 2004a). Transdermal estrogen replacement therapy has been known to be effective for patients with depressive symptoms during the perimenopausal period (Soares et al., 2001). In this way, there are various arguments about perimenopausal depression. However, it’s certain that sex hormones changes and also consequent stresses occur during the perimenopausal period, and this with addition of factors such as aging, the increase of physical illness, the changing role of women in society and family, and so on, the risk for depression during perimenopausal period may increase. The association between the estrous cycle and depression is probably bidirectional and interactive, making it difficult to understand the mechanism of the pathogenesis of perimenopausal. A history of major depression may be correlated with early onset of menopause due to an early diminution of ovarian function (Harlow et al., 2003). Studies in animal experiments frequently adopt an ovariectomy model, but surgically induced menopause after ovariectomy cannot be always identified with natural menopause. These facts make it difficult to research the mental condition during perimenopausal period.

#### Other psychiatric disorders associated with sex differences

Sex hormonal differences may also be related to some other psychiatric disorders; for example, autism including adult Autism Spectrum Disorders (ASD) is more common in men than women (Brugha et al., 2011). Schizophrenia occurs with equal rates in both sexes, but it has been widely known that schizophrenia in women occurs in older ages and female patients with schizophrenia have better prognoses than men with schizophrenia. The plasma level of testosterone in male patients with bipolar disorder has been reported to be positively correlated with the number of manic episodes and the number of suicide attempts (Sher et al., 2012). These reports suggest the importance role of sex hormones in psychiatric disorders. So far, a great number of studies have been conducted with the traditional view of sex hormones acting mainly on neurons and neurotransmitters, but a few recent studies have highlighted the association between the sex hormones and microglia.

### Sex hormones and microglia

The relationship between sex hormones and microglia has not been well understood. However, some studies have recently presented interesting facts about the relationship.

#### Estrogen

Several investigations suggest that some sex hormones have anti-neuroinflammatory and neuroprotective activities via microglia. *In vitro* studies using rat microglial cells have revealed that estradiol inhibits phagocytosis, the production of ROS (Bruce-Keller et al., 2000), and LPS-induced pro-inflammatory molecules such as inducible nitric oxide synthase (iNOS), prostaglandin E2 (PGE2), and matrix metalloproteinase-9 (MMP-9) (Vegeto et al., 2001). The expression of rat microglial neuroinflammatory genes by the immunosuppressive functions of estradiol is mediated via estrogen receptor alpha (ERα) and beta (ERβ) (Sarvari et al., 2011).

It is important to investigate the response of microglia to various pro-inflammatory stimuli from the perspective of age and sex differences. Morphology and numbers of microglia alter during developmental stages. For instance, there are more microglia derived from male postnatal rats during an early stage of development than female, and the shapes of microglia derived from female postnatal rats during later developmental stages tend to be amoeboid type (Schwarz and Bilbo, 2012). The distinct microglial expression of genes of cytokines, chemokines, and receptors are attributed to age and sex differences (Schwarz and Bilbo, 2012). Similarly, expression of P2 purinergic receptors varies with age and sex in murine microglia (Crain et al., 2009). Estradiol has proved to play distinct roles depending on the situation. For instance, in female neonatal rats, microglia express more IL-1β with estradiol *in vitro*, that is to say, estradiol exerts a pro-inflammatory effect on female microglia, and on the contrary, estradiol has an anti-inflammatory effect on male microglia. Moreover, in adult rats, estradiol has pro-inflammatory effects without sex differences *ex vivo* (Loram et al., 2012). Chronic administration of estradiol *in vivo* results in the activation of microglia derived from the female hippocampus inducing IL-1β expression stimulated with LPS (Loram et al., 2012). These reports have suggested that estradiol plays different roles in modulating microglia depending on age and sex.

A remarkable function of estrogen receptor on microglia has recently been elucidated. 5-androsten-3b,17b-diol (ADIOL), converted from dehydroepiandrosterone by 17b-hydroxysteroid dehydrogenase type 14 (HSD17B14), has been proved to suppress inflammatory responses of microglia and astrocytes by the recruitment of C-terminal binding protein (CtBP) through an ERβ dependent mechanism (ADIOL/ERβ/CtBP transrepression pathway in microglia). Moreover, through this pathway, the administration of ADIOL has prevented and inhibited experimental autoimmune encephalomyelitis (EAE), an animal model of multiple sclerosis (MS) (Saijo et al., 2011).

#### Progesterone/testosterone

Progesterone is widely known to play an important role in inflammation in the peripheral organs and the CNS (Stein, 2001; Brinton et al., 2008; Challis et al., 2009). Progesterone and testosterone also regulate microglial functions. Progesterone antagonizes estradiol in synaptic remodeling, which is mediated by a progesterone receptor on microglia in rats (Wong et al., 2009). Brain injury increases the expression of aromatase that metabolizes testosterone to estradiol, and the administration of testosterone reduces the number of astrocytes and microglia in the lesion (Barreto et al., 2007).

#### Prolactin

Prolactin is a sex-related hormone. Prolactin is mainly synthesized by and secreted from the anterior lobe of pituitary gland. The secretion of prolactin is mediated by prolactin inhibiting factors, predominantly dopamine (Ben-Jonathan and Hnasko, 2001), and prolactin releasing factors such as TRH and oxytocin (Egli et al., 2010). Other factors stimulating the secretion of prolactin include suckling and various stresses (Otte et al., 2002; Lennartsson and Jonsdottir, 2011). Prolactin has multiple functions such as lactation, maintenance of gestation, mediating maternal behavior (Freeman et al., 2000), and has been suggested to have anxiolytic and anti-stress effects on the evidence of an experiment with rats (Torner et al., 2001).

Möderscheim et al. have just reported the first possible correlation between prolactin and microglia. They have shown that unilateral hypoxic ischemic injury in rats made astrocytes and reactive microglia strongly prolactin immunoreactive, and prolactin immunoreactivity was increased in the affected cortex prolactin. On the other hand, prolactin and prolactin receptors were decreased on penumbral neurons. In this report, prolactin has been proved to be proliferative for astrocytes *in vitro*, but it has been uncertain whether prolactin has any effect on microglia (Möderscheim et al., 2007).

### Clinical implications

**Figure 2:**
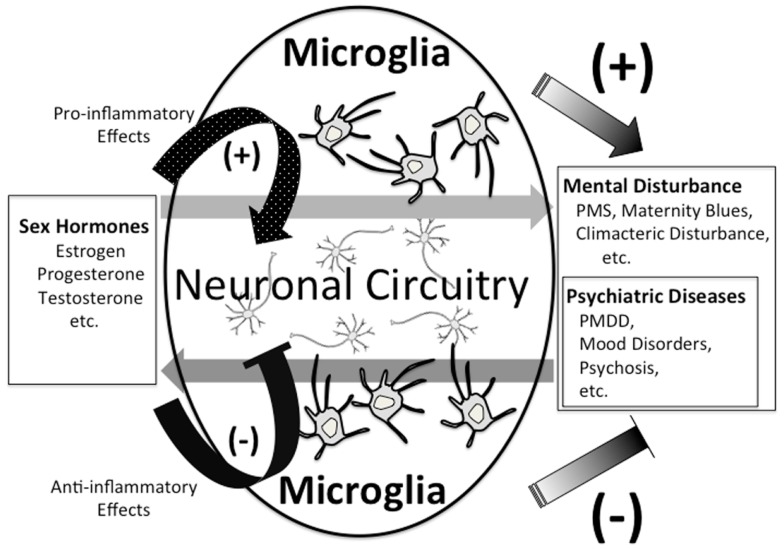
**Possible link between sex hormones and microglia**.

As stated above, we are just in the early stages of digging up the relationship between sex hormones and microglia. The role of sex hormones in neuropsychiatric disorders such as MDD, PMDD, and postnatal depression is being gradually clarified. The existence of sex differences in a variety of physical diseases has also been widely known, and sex hormones play crucial roles in the pathophysiology of autoimmune diseases, such as MS, systemic lupus erythematosus, and rheumatoid arthritis which are reported to be more prevalent in women than in men (Duquette et al., 1992; Ostensen et al., 2011). In contrast to this prevalence, women with MS have better prognoses than men, and men with MS show more progressive disease course and more severe gray matter atrophy (Voskuhl and Gold, 2012). Both the incidence and activity of MS decrease during the latter half of pregnancy (Confavreux et al., 1998), and increase in the postpartum period (Alonso et al., 2005; Finkelsztejn et al., 2011). In addition, it has been reported that the experience of more pregnancies increases the rate of MS onset (Ponsonby et al., 2012). These reports imply a significant role for sex hormones in the pathogenesis of MS. It is widely know that microglia also play an important role in MS (Jack et al., 2005). Regarding the pathogenesis of some psychiatric diseases involved with sex differences, the interactive network where various psychiatric diseases interact with sex hormones, the fluctuation of sex hormones, and the vulnerability to the fluctuation may be involved. Neurons have been traditionally considered to be the main player in such interactive networks, which have been studied with an emphasis on various neurotransmitters, for example, glutamate (Batra et al., 2008), serotonin (Roca et al., 2002; Hiroi et al., 2006; Dhingra et al., 2007; Landen et al., 2007; Brown et al., 2009), and GABA (Epperson et al., 2002). We hypothesize that not only neurons but also microglia may play an important role as a connector in the network. Sex hormones such as progesterone may develop and aggravate psychiatric disorders via microglial inflammatory responses through their receptors. On the other hand, sex hormones such as estrogen and androgen suppress microglial inflammatory responses through their receptors, which may induce therapeutic effects in psychiatric disorders (Figure 2). In order to profoundly understand the relationship between sex hormones, psychiatric disorders, and microglia, it is necessary to investigate how sex hormones and microglia directly interact, and also the microglial behavior in the above-mentioned networks.

## Oxytocin

Oxytocin is secreted by the posterior pituitary gland and synthesized in the paraventricular and supraoptic of the hypothalamus (Moos et al., 1984). Oxytocin, which is released within the supraoptic nucleus of hypothalamus, exerts positive feedback on the production and release of oxytocin into the peripheral circulation and the CNS (Neumann et al., 1994, 1996). Oxytocin has been classically known as the hormone that is related to uterine contraction, lactation, and the regulation of atrial natriuretic peptide (ANP) release in the cardiovascular system (Jankowski et al., 1998, 2000). Besides, oxytocin is released into the CNS and facilitates maternal nurturing behavior, the bondage between a mother and child, and affiliative behavior and social cognition in both sexes (Ross and Young, 2009). Recently, the intranasal administration of oxytocin in humans proved that oxytocin is a possible prosocial hormone that increases trust (Kosfeld et al., 2005). In addition to these functions, oxytocin is associated with stress-suppression effects. In rats, the administration of a SSRI has shown to increase the plasma concentration of oxytocin (Uvnas-Moberg et al., 1999). In humans, the intranasal administration of oxytocin has been reported to exert an anxiolytic effect on healthy men, and reduce salivary concentration of cortisol in combination with social support in response to stress (Heinrichs et al., 2003). It is controversial whether the plasma concentration of oxytocin is low in patients with depression, but the negative correlation between plasma oxytocin concentration and scores of Hamilton Depression Rating Scale (HDRS) and the State-Trait Anxiety Inventory (STAI)/A-trait among depressed patients has been reported (Scantamburlo et al., 2007). A human postmortem study has revealed that the number of oxytocin neurons increases in the PVN of the hypothalamus in patients with MDD or bipolar disorder (Purba et al., 1996). These reports suggest various functions of oxytocin in human mental activities.

### Oxytocin and autism

Recent studies have suggested that oxytocin is probably relevant to psychiatric disorders, especially autism. The intravenous administration of oxytocin to autistic patients increases retention of social cognition (Hollander et al., 2007), on the other hand, the intranasal administration of oxytocin ameliorates emotional cognition. Oxytocin inhalation strengthens social interactions, induces more adaptable social behavior, and enhances feelings of trust and preference (Andari et al., 2010). However, the underlying mechanism explaining how oxytocin exerts these effects on autism has not been sufficiently clarified. The relationship between autism and a variation in the CD38 gene has been reported (Munesue et al., 2010). CD38, a multi-functional molecule, is involved in the secretion of oxytocin. Amelioration of neural processing of social stimuli by the intranasal administration of oxytocin depends on a CD38 gene variant in healthy volunteers (Sauer et al., 2012). Female CD38 knockout mice defect in maternal nurturing, and male knockout mice impair prosocial behavior (Jin et al., 2007). Observed in oxytocin receptor knockout mice, the intracerebroventricular administration of oxytocin and vasopressin (AVP) ameliorates impaired cognitive flexibility, social deficits, increased aggression, and seizure susceptibility, which is mediated by an AVP receptor (V1a) (Sala et al., 2011). These reports have suggested that oxytocin has a variety of psychotropic, prosocial, and subsequent therapeutic effects in animal models of autistic disorders, but in humans with autism, whether oxytocin has therapeutic effects have not been sufficiently validated yet so far, and needs to be further investigated.

### Autism and microglia

Microglia may play an important role in autism. Rett syndrome is classified as an ASD, mainly caused by *X*-linked methyl-CpG-binding protein 2 (MeCP2) gene mutations (Amir et al., 1999). MeCP2 gene is expressed predominantly in neurons in the CNS and prompts neuronal differentiation (Tsujimura et al., 2009), indicating that the etiology of Rett syndrome mainly caused by MeCP2 gene mutations is of neuronal origin (Chen et al., 2001). On the other hand, it has been recently suggested that glial cells such as astrocytes and microglia are also involved with the pathology of Rett syndrome (Maezawa et al., 2009; Maezawa and Jin, 2010). In a mouse model of Rett syndrome, transplantation of wild-type bone marrow resulted in wild-type microglial engraftment, which showed significant improvements in autistic symptoms (Derecki et al., 2012). A postmortem study on the brains of autistic patients revealed an increase of microglia in the fronto-insular cortex and visual cortex (Tetreault et al., 2012). Moreover, a spatial pattern analysis of the postmortem brains of autistic patients indicated the abnormal interaction between microglia and neurons especially in the dorsolateral prefrontal cortex (Morgan et al., 2012). These postmortem studies of child autism have suggested that the inflammatory hypotheses of psychiatric disorders are, to some extent, rooted in unique periods of vulnerability when an inflammatory assault permanently re-wires the brain for the expression of these neurodevelopmental disorders. A recent PET study has shown that excessive microglial activation in multiple brain regions has been observed in young adult subjects with ASD (Suzuki et al., 2013). These reports suggest that microglial activation during adulthood may moderate the disease symptomatology of autism, while it is unclear whether microglial activation has lasted from childhood or not.

### CD38, oxytocin, and microglia

To the best of our knowledge, there are only a few studies about the relationship between CD38, oxytocin, and microglia. CD38, a multi-functional molecule involved in oxytocin secretion, has some effects on microglia. Firstly, Mayo et al. (2008) have reported that CD38 generates cyclic-adenosine diphosphate ribose (cADPR) from nicotinamide adenine dinucleotide (NAD) as a substrate. CD38, via cADPR as a second messenger, increases intracellular calcium concentration and helps to promote microglial activation and activation-induced cell death (AICD) in primary mouse microglia induced by LPS/IFN-γ treatment. Secondly, Ma et al. (2012) have reported that siRNA for CD38 gene silencing promotes caspase 3-dependent apoptosis, which decreases survival of BV2 microglia cells. Oxytocin also interacts with microglia. Karelina et al. (2011) have reported that oxytocin suppresses LPS-induced expression of MHC class II dose-dependently in primary mouse microglia.

### Perspective

Microglia and CD38 may possibly play crucial roles in the pathophysiology and treatment of autism. Oxytocin has a variety of effects on autistic patients (Hollander et al., 2007; Andari et al., 2010; Sauer et al., 2012) and stress-suppression effects as mentioned above, which implies a yet to be fully comprehended relationship between oxytocin and microglia. However, there have been so few reports about the relationship that we can only be certain of the following two points; one, that unknown factors secreted by microglia inhibit the oxytocin receptor binding in astrocytes (Mittaud et al., 2002), and two, that macrophages, close-related with microglia, express oxytocin receptors and are inhibited by oxytocin from secreting LPS-induced Interleukin-6 and decreasing NADPH-dependent superoxide activity (Szeto et al., 2008). A recently published *in vitro* study also showed pro-inflammatory effects of oxytocin on activated macrophage (Oliveira-Pelegrin et al., 2013). It is unclear and yet to be investigated whether microglia could be involved with production and release of oxytocin via direct or indirect action on the hypothalamus or the pituitary gland, and the interaction between oxytocin and microglia should be clarified in the future. In addition, to our knowledge, we are not aware whether the intranasal administration of oxytocin is physiologically comparable to hormonal levels released by microglia or not, and this aspect should be investigated in future research.

## Final Remarks; Missing and Possible Links between Neuroendocrine Factors, Neuropsychiatric Disorders, and Microglia

Neuropsychiatric disorders have been mainly considered as the consequence of abnormal conditions in neural circuitry. Many neuropsychiatric disorders are widely known to be involved with endocrine diseases, however the correlation has not been well understood. Microglia have been suggested to have several important roles in neuropsychiatric disorders. We have introduced up-to-date knowledge on the interaction between neuroendocrine factors, neuropsychiatric disorders, and microglia; especially highlighting the hormones; CRH, glucocorticoids, estradiol, and oxytocin. We believe that some microglial roles may be revealed by further research efforts in line with our proposed hypothesis. Finally, we have shown some possible mechanisms and missing links to understand the interaction between neuroendocrine factors, neuropsychiatric disorders, and microglia.

### Possible mechanism

**Figure 3:**
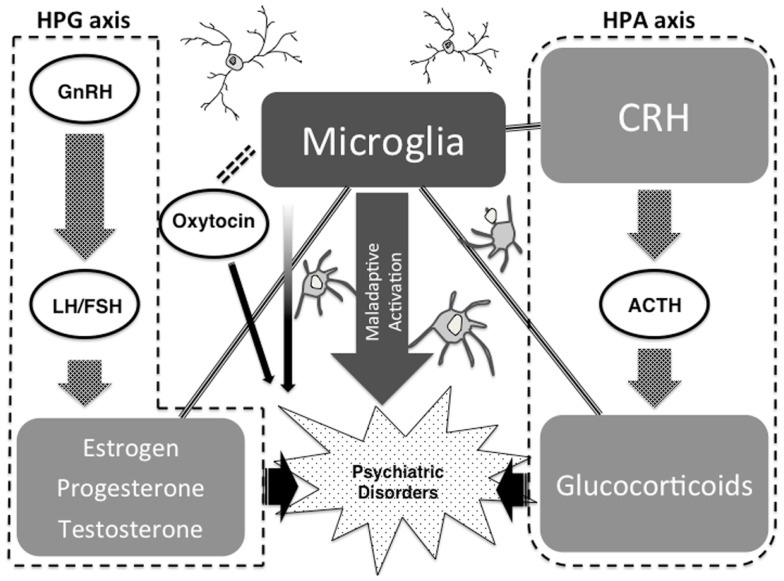
**Possible link between neuroendocrine factors, neuropsychiatric disorders, and microglia**.

Strong psychological stress and/or physical stress induce neuroendocrine hormones such as CRH in the brain, which may lead to microglial activation. Especially during childhood, microglial over-activation due to severe stress may formulate maladaptive neuronal networks due to a microglial impacted synaptic pruning. A recent study has shown the novel role of NO as an activity-dependent regulator of target neuron intrinsic excitability, transforming synaptic integration and information transmission (Steinert et al., 2011), and we suppose that microglia-derived NO may contribute to this phenomenon. The above highlighted maladaptive synaptic formulations themselves may induce psychiatric conditions such as autistic syndrome. On the other hand, when these maladaptations are not severe, small pathologies may be reserved as vulnerabilities for psychiatric disorders such as schizophrenia and depression in later life. Stressful life events during adolescent periods and adulthood, as a secondary hit, may also activate the above-mentioned maladaptive cascades of neuroendocrine-microglia-neuronal networks, which may induce psychiatric conditions. Based on evidence that we have introduced, we hypothesize that microglia and microglia-derived mediators such as inflammatory cytokines and free radicals may be a bridging player between our highlighted neuroendocrine factors and psychiatric disorders (Steiner et al., 2011; Maes et al., 2012) (Figure 3).

### Missing and possible link between HPA and HPG axis

Endocrine systems such as the HPA axis and the HPG axis communicates with the other, and these mutual interactions play important roles in neuropsychiatric disorders (Tsigos and Chrousos, 2002; Ochedalski et al., 2007; Pittman, 2011; Schwarz and Bilbo, 2012). Many reports have indicated that the HPA axis and HPG axis interact with each other in various ways. Concentration of cortisol in plasma has been highly maintained during the third trimester of normal pregnancy (Nolten et al., 1980), probably due to high concentration of estrogen and progesterone (Bloch et al., 2003). The concentration of CRH decreases due to the negative feedback system during the third trimester of pregnancy, and the low concentration of CRH remains for a period of time and causes the hypoactivity of the HPA axis after delivery. In patients with postpartum depression, this period of HPA axis hypoactivity is maintained longer than in healthy puerperant women (Magiakou et al., 1996). Cortisol level of patients with postpartum depression does not respond to the administration of ACTH, suggesting the HPA axis dysregulation during the postpartum period (Jolley et al., 2007). Bloch et al. have reported an abnormal response to the alteration of sex hormones in patients with postpartum depression. They have shown the increased reactivity of cortisol to the administration of CRH in euthymic patients with a past history of postpartum depression (Bloch et al., 2005), and that the administration of a high dose sex hormone followed by abrupt withdrawal induce depressive symptoms (Bloch et al., 2000). Thus, the HPA axis may be involved with the pathogenesis of postpartum depression. HPA axis activation has been known to suppress the HPG axis (Petraglia et al., 1987; Polkowska and Przekop, 1997). It is necessary to investigate the yet to be clarified microglial role in the dynamic interactions between the HPA and HPG axis.

### Possible example

It is epidemiologically well known that suicide has a higher prevalence in men, and is lower in women. This review paper has indicated that progesterone/testosterone (so-called male hormones) may induce maladaptive microglial activation, and estradiol (so-called female hormone) may suppress microglial inflammatory reactions. A significant number of studies suggest that testosterone is associated with aggression, and aggression is positively linked to suicidal behaviors (Sher, 2012). In addition, a recent postmortem study has shown the positive link between microglial activation and suicide (Steiner et al., 2006, 2008). Summing up such cellular, clinical, and epidemiological evidence, we have proposed a possible mechanism – that male hormones may easily induce suicidal acts by hormone-induced microglial activation, and estradiol may prevent suicidal behaviors by suppressing microglial activation.

### Limitations and future perspective

Endocrine factors such as steroid hormones may interact with microglia to produce inflammation-dependent neuropsychiatric conditions. If so, what signals allow for discrimination between schizophrenia, autism, and depression? If microglia are the common link between neuroendocrine systems and neuropsychiatric disorders, then what differentiates the role of microglia in these disorders? These research questions have yet to be clarified and should be focused on in future research. As reviewed above, each hormone affects microglia differently in different brain regions, which might be a cue to explore the dark-side mechanism. A growing body of evidence has revealed that abnormalities of specific brain regions contribute to each neuropsychiatric disorder and each psychiatric condition. We suppose that the location of the microglia-induced neuropathology may determine the specific psychiatric abnormality. On the other hand, not only microglia but also other brain cells such as brain macrophage, T cells, and astrocytes are known to release cytokines, chemokines, and free radicals. Therefore, these alternative pathways may also be important in the process.

Future investigations to clarify the correlation between neuroendocrine factors and microglia may contribute to a novel understanding of the pathophysiology of neuropsychiatric disorders and the development of effective treatment strategies.

## Conflict of Interest Statement

This work was financially supported by Grant-in-Aid from the Japan Society for the Promotion of Science (JSPS) to Takahiro A. Kato and Shigenobu Kanba. All the authors have declared that no competing interests exist.

## Abbreviations

ACTH, adrenocorticotropic hormone; ADIOL, 5-androsten-3b,17b-diol; ASD, autism spectrum disorders; AVP, vasopressin; cADPR, cyclic-ADP ribose; CRH, corticotropin-releasing hormone; CRH-R1, CRH receptor I; CRH-R2, CRH receptor II; CtBP, C-terminal binding protein; ER, estrogen receptor; GABA, gamma-aminobutyric acid; HPA axis, hypothalamic-pituitary-adrenal axis; HPG, hypothalamic-pituitary-gonadal; IFN-γ, interferon gamma; IL, interleukin; LPS, lipopolysaccharide; MeCP2, *X*-linked methyl-CpG-binding protein 2; MS, multiple sclerosis; NO, nitric oxide; O&NS, oxidative and nitrosative stress; PMDD, premenstrual dysphoric disorder; PTSD, post-traumatic stress disorder; PVN, paraventricular nucleus; ROI, reactive oxygen intermediates; ROS, reactive oxygen species; TNF-α, tumor necrosis factor-α.
